# Does the Anchor Eye Alignment Affect the Performance of the Rotator Cuff Repair? A Biomechanical Study

**DOI:** 10.1016/j.asmr.2020.10.005

**Published:** 2021-02-01

**Authors:** Lara Locatelli, Cesar A.Q. Martins, Arthur P.G. Santos, Patricia O. Cubillos, Carlos R.M. Roesler

**Affiliations:** a Governador Celso Ramos Hospital, Florianópolis, Brazil; b Serviço de Ortopedia e Traumatologia, Sinop, Brazil; c Laboratory of Biomechanical Engineering, University Hospital, Department of Mechanical Engineering, Federal University of Santa Catarina, Florianópolis, Brazil

## Abstract

**Purpose:**

To investigate the metal screw-in anchor failure mode and load to failure for 2 different eyelet alignments after anchor insertion in ovine humeri.

**Methods:**

Sixteen ovine humeri were dissected, and a 5-mm metal anchor with 2 nonabsorbable polyblend polyethylene sutures was inserted into them in the proximal position of the greater tuberosity. The alignment of the anchors after insertion was adjusted to make 2 test groups, each with 8 specimens: In group 1, the anchor eyelets were malpositioned, whereas in group 2, the anchor eyelets were aligned according to the manufacturer's instructions. After insertion, cyclic tests from 10 N to 180 N were performed with a frequency of 1 Hz for 200 cycles; specimens were then loaded to failure to evaluate the maximum load of the system and observe the associated failure mode.

**Results:**

The mean ultimate failure load in group 2 was not significantly different from that in group 1 (*P* = .472).

**Conclusions:**

For metallic screw-in suture anchors, the alignment of the eyelet does not change the failure mode and the load to failure after cyclic loading of the bone-anchor-suture system in ovine humeri.

**Clinical Relevance:**

Our results indicate that on the basis of this anchor model, the position of the eyelet in the greater tuberosity does not interfere directly with the biomechanical performance of the system.

The use of implants in rotator cuff repair has changed dramatically in the past few decades, and the use of anchors was the most important innovation after the advent of arthroscopic shoulder surgery.^1^ Several types of anchors are currently available, varying in material (metallic or polymeric), size, and design. During development, these products must be experimentally tested to validate the design and to verify that the finished product meets all the safety and effectiveness requirements. The main parameters evaluated refer to the pullout force and failure mode.

Previous studies have evaluated the angle of insertion of the anchor into the greater tuberosity, with Burkhart's “deadman” theory being the most accepted theory today. However, little is known about the different alignments that the anchor eyelet can assume after the anchor is inserted into the bone. In fact, when eyeleted anchor designs are used, the plane containing the hole of the anchor eyelet may be positioned parallel to the sagittal plane of the humerus or lie perpendicular the sagittal plane of the humerus. Most implant manufacturers provide an insertion guide so that the plane containing the hole of the eyelet lies perpendicular to the sagittal plane of the humerus.^2^ In this position, the suture wire tension is supported by the rounded edge of the eyelet.

The purpose of this study was to investigate the metal screw-in anchor failure mode and load to failure for 2 different eyelet alignments after anchor insertion in ovine humeri. The hypothesis was that there would be a difference in the ultimate failure load and associated failure mode between the 2 alignments because the contact of the suture with the inner surface of the eyelet is not the same.

## Methods

### Anchor Systems

Sixteen Titanium-6Aluminum-4Vanadium alloy 5.0-mm metallic anchors (ASTM F136) of the same model and manufacturer (Hexagon Indústria e Comércio de Implantes Ortopédicos, Itapira, Brazil) were used in this study. Each anchor was loaded with 2 nonabsorbable polyblend polyethylene sutures (No. 2 FiberWire; Arthrex, Naples, FL). Polyblend sutures were used because of their superior mechanical properties to the frequently used polyester sutures, and they were made by L.L.^3^, ^4^, ^5^

### Eyelet Surface Analysis

Because the inner surface of the eyelet has direct contact with the suture wire, any defects of the eyelet surface may cause a rupture of the suture. So, before the biomechanical tests, the inner surface of the eyelet of each anchor was inspected by scanning electron microscopy to identify any defects that could negatively impact the behavior of the wire-eyelet interface. The analyses were performed in a scanning electron microscope (model JSM-6390LV; Jeol, Akishima, Japan), with a power of 15 kV, using a secondary electron detector, at various magnifications.

### Bone Model

Sixteen fresh-frozen ovine shoulders were stripped of all soft tissue by an experienced shoulder surgeon (L.L.). The location for insertion of the anchor was defined after decortication. The specimens were frozen immediately after harvesting and were thawed for 24 hours at room temperature before testing and screened for osteoarthritic changes and deformities. No specimens were excluded during testing. Ovine shoulders were chosen as the bone model because healthy ovine and human bones have similar failure loads when testing suture anchors.^6^ Moreover, ovine bones have been used in several previous studies for anchor evaluations in rotator cuff repair.^7^, ^8^, ^9^, ^10^

### Anchor Fixation

The fixation of the anchors was carried out by an experienced board-certified shoulder surgeon (L.L.) using the arthroscopic ancillary dedicated to the anchor’s insertion. The insertion site in the ovine model was the proximal-middle region of the greater tuberosity to simulate the force vector in an ideal human insertion site more accurately.^11^ The proximal-middle part has a significantly higher cortical bone mineral density in humans than the other parts of the greater tuberosity, and the pullout strength of the suture anchors is significantly affected by cortical bone mineral density.^9^ The anchors were then inserted 45° to the bone surface (deadman angle), maximizing the strength of anchor fixation as described by Burkhart,^12^ with the eyelet flush with the bone. A specially built insertion guide was used to reproduce the angle of introduction for all ovine shoulders in a consistent manner.

For the biomechanical comparison of different alignments of the eyelet, the anchors were divided into 2 groups: In group 1 (n = 8), the plane of the hole of the anchor eyelets was positioned parallel to the humeral sagittal plane. In group 2 (n = 8), the anchors were positioned according to the manufacturer’s orientation, in which the plane of the hole of the eyelets was positioned perpendicular to the humeral sagittal plane.

### Mechanical Testing

**Fig 1:**
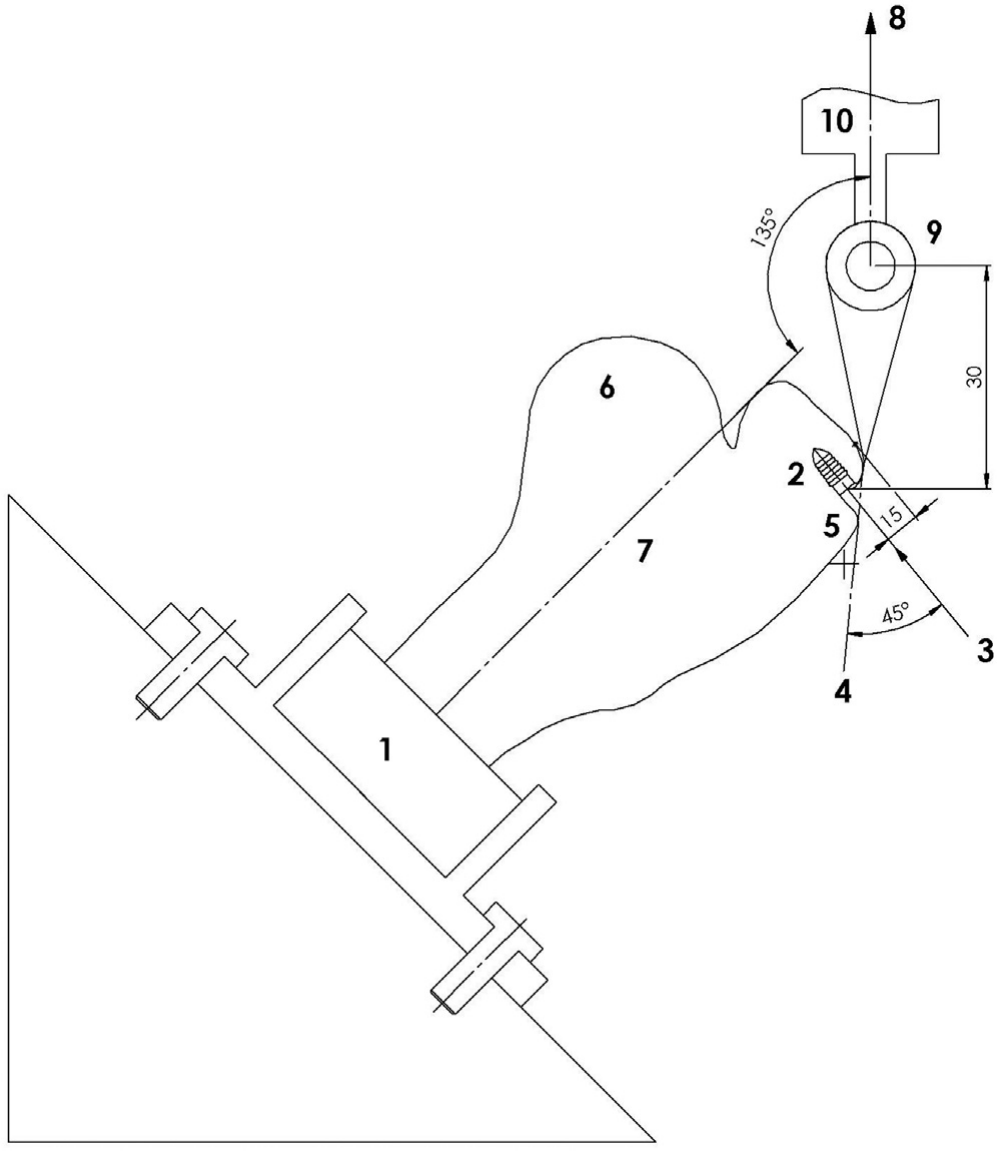
Fixation device: proximal part of humerus (1), bone anchor (2), direction of insertion of anchors (3), tangential line (4) over greater tuberosity (5), superior part of humeral head (6), axis of humeral shaft (7), direction of pullout tests (8), bar for fixation of sutures (9), and crosshead of testing machine (10).

Immediately after anchor fixation, each humeral shaft was clamped to a custom device (fixture) with bone cement (polymethyl methacrylate). This device was then placed in the servo-hydraulic testing machine (MTS Bionix model 370.02; MTS Systems, Minneapolis, MN) ensuring an angulation of 135° between the humeral axis and loading direction, re-creating the vector of force that would occur after a rotator cuff repair.^7^ The free end of the wire was fixed with a surgeon’s knot in the load cell, leaving a gage length of 30 mm between the knot and the anchor, as shown in Fig 1. The knot was made by an experienced board-certified shoulder surgeon (L.L.). A pre-tension of 10 N was applied to the specimen.

### Cyclic Loading

Each specimen was cycled from 10 N to 180 N at 1 Hz for 200 cycles, held to 10 N for 5 seconds, and then loaded to failure at 33 mm/s. These parameters have been reported as the physiological loads and speeds that occur in normal daily activity and were similar to those used in prior studies.^13^, ^14^, ^15^ The parameter recorded in cyclic tests was displacement after the first cycle at 180 N, as well as after 200 cycles, to evaluate laxity during the cyclic load, related to loss of fixation stability with delayed healing. The ultimate failure load and the failure mode of the system were registered in the load-to-failure test. Failure during cyclic loading or load-to-failure testing was assumed to occur in cases in which complete slippage of the fixation device or complete pullout of the anchor or rupture of the wire was observed. Biomechanical tests were filmed to analyze the rupture site. After frame-by-frame observation of each test, the location of the rupture was classified as either at the eyelet or not at the eyelet (suture breakage).

### Statistical Analysis

A power analysis was conducted after a pilot study of 12 specimens (6 per group) using G∗Power 3 for Mac software (Heinrich-Heine-Universität Düsseldorf, Dusseldorf, Germany) for failure load, displacement after the first cycle, and displacement after the 200th cycles. A total of 16 specimens (8 in each group) were needed to achieve a minimum of 80% power at a 1-sided 5% significance level.

Data analysis and calculation of statistical significance were performed with R software (version 3.5.2; R Foundation for Statistical Computing, Vienna, Austria). Considering normal data distribution, we used the parametric Welch *t* test to evaluate the difference between mean failure load and displacement at the 200th cycle. The displacement data at the first cycle failed on the Shapiro-Wilk normality test; therefore, the Wilcoxon test was used to assess the mean displacement. The Fisher exact test was used to evaluate failure modes. *P* < .05 was considered significant.

## Results

**Fig 2:**
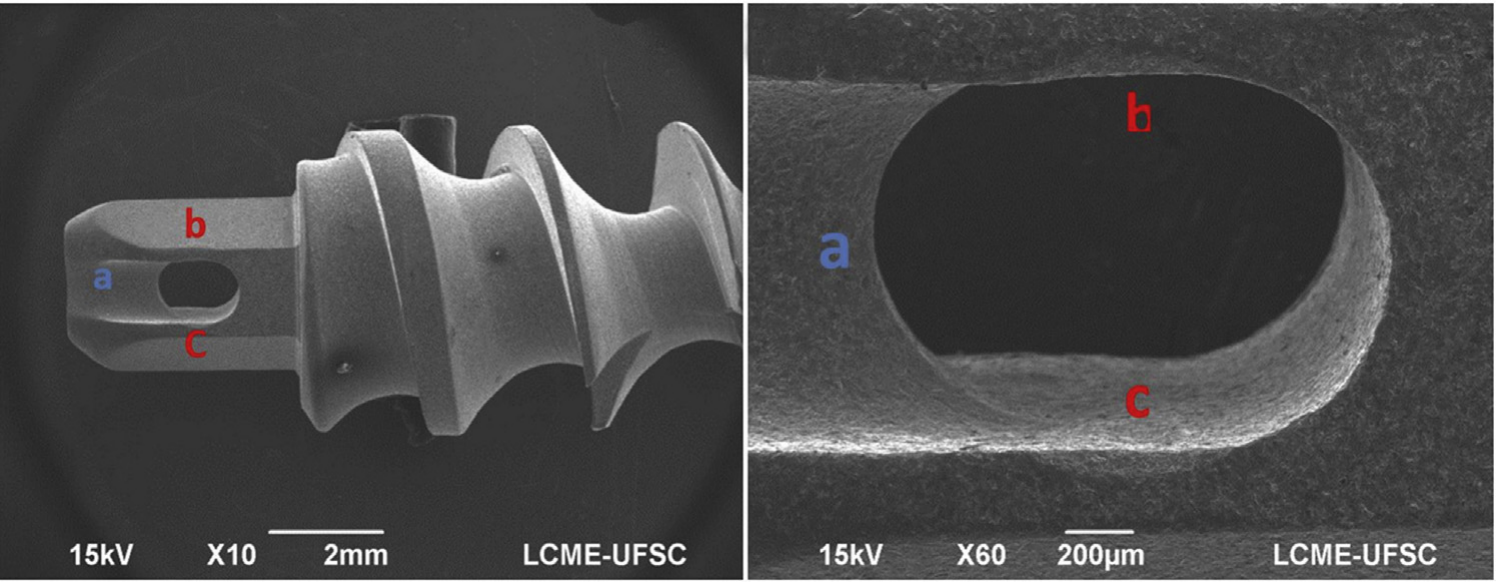
Scanning electron microscopy: regions of eyelet contact with FiberWire seen from same anchor. The region in contact with the wire in group 2 is indicated by a. The regions in contact with the wire in group 1 are indicated by b and c (zoomed in). (LCME, Laboratório Central de Microscopia Eletrônica UFSC.)

The mean ultimate failure load of the system (in newtons), displacement after the first cycle and after the 200th cycle (in millimeters), and failure modes are reported in Table 1, together with the standard deviations. The regions of the eyelet that come into direct contact with the suture were analyzed in 2 positions of the anchor tested in relation to the sagittal plane of the humerus (Fig 2). Analysis of the scanning electron macrographs showed that none of the anchors had any type of defect on the inner surface of the eyelet that could negatively influence the performance of the mechanical test or generate the rupture of the suture.

**Table 1:** Comparative Results of Biomechanical Tests

Variable	Group 1 (n = 8)	Group 2 (n = 8)	*P* Value
Failure load, N	350 ± 67	373 ± 56	.472∗
Displacement after first cycle, mm	6.11 ± 0.64	5.51 ± 1.20	.188†
Displacement after 200th cycle, mm	7.3 ± 0.79	7.03 ± 1.01	.574∗

NOTE. Data are given as mean ± standard deviation. Data for the different test groups include failure load, displacement after the first cycle, and displacement after the 200th cycles. In group 1, the plane of the hole of the anchor eyelet was positioned parallel to the humeral sagittal plane. In group 2, the anchor was positioned with the plane of the hole of the eyelet perpendicular to the humeral sagittal plane.

∗ Parametric Welch *t* test.

† Nonparametric Wilcoxon test.

The loads up to system failure ranged from 229 to 459 N in group 1, with a mean of 350 N. For group 2, the failure load ranged from 290 to 478 N, with a mean of 373 N (Table 1). Load-to-failure values did not show a statistically significant difference between the groups tested (*P* = .472).

After the first cycle, both groups had a maximum displacement of 7.0 mm, with mean values of 6.11 mm in group 1 and 5.51 mm in group 2. These displacement values did not present a statistically significant difference between the tested groups (*P* = .188). After the last cycle, group 1 presented a maximum displacement equal to 8.2 mm and a mean value of 7.3 mm (1.19 mm of elongation) whereas group 2 showed a maximum displacement equal to 8.7 mm and a mean value of 7.03 mm (1.52 mm of elongation). These displacement values did not present a statistically significant difference between the tested groups (*P* = .573).

Concerning the failure mode (Table 2), there was no statistically significant difference between the tested groups (*P* = .619). Therefore, the eyelet alignment appears to have no influence on the location of the suture rupture.

**Table 2:** Comparative Results for Failure Mode

Variable	Group 1 (n = 8), n	Group 2 (n = 8), n	*P* Value
Rupture at eyelet	5	3	.619
Rupture not at eyelet	3	5	

NOTE. The failure mode was considered the site of rupture: either at the eyelet (i.e., rupture of the suture occurred at the eyelet) or not at the eyelet (i.e., rupture occurred on the FiberWire but not at the eyelet). The Fisher exact test was used for a 2 × 2 contingency table to test the difference in failure mode between the groups.

## Discussion

For the 2 eyelet alignments investigated, the mean pullout force was not statistically significantly different when the suture was in contact with the rounded surface of the eyelet. The 2 groups also showed no statistically significant differences in failure mode. In group 1, in which the suture was in contact with the straight inner surface of the eyelet, the predominant failure mode was suture rupture inside the eyelet (5 cases), whereas in group 2, the predominant failure mode was suture rupture but not at the eyelet (5 cases). Despite this finding, statistical analysis showed that the biomechanical performance of both groups was not different in this investigation.

To evaluate the eyelet-suture interface in-depth, electronic microscopy was performed on all the anchors before being inserted into the humerus. The anchor eyelet is in direct contact with the suture, and therefore, any imperfection in the eyelet could dictate the failure mode in the mechanical tests. On analysis of the macrographs performed on the scanning electron microscope, no type of defect or irregularity was found on the inner surface of the eyelets, and therefore, the results of the mechanical tests were not influenced by any imperfections of the eyelets.

In the literature, only 3 studies were found in which the anchor-bone-wire system was tested in the same way as in our study.^7^^,^^9^^,^^16^ In most of the published studies that tested the primary anchor fixation force in bone, the tests were performed with the force vector parallel to the anchor axis and with the same inserted at 90° to the bone, differing from clinical practice.

Pietschmann et al.^16^ performed a comparison between transosseous sutures and anchors in rotator cuff repair, assessing tear strength and failure mode, with the force vector at 135° to the long axis of the humerus. They tested 3 types of anchors in cadaveric humeri but only 1 metal model, similar to that used in our study. The mean failure load was 188 N in non-osteoporotic bone, a mean force much lower than the values found in this study. Regarding the failure mode, the most common cause was wire breakage in the eyelet (4), followed by a rupture of the wire outside the eyelet (1) and anchor pullout (1).

Schneeberger et al.^8^ tested several anchors in cadaveric humeri. They evaluated the failure mode and maximum load to failure using the force vector of 135° to the diaphyseal axis. Among the anchors tested, only 1 was similar to the anchor used in our work. The maximum load to failure was 140 N, and the failure mode was suture breakage in the eyelet (4) or anchor pullout (1). De Carli et al.^17^ tested a metal anchor model similar to that used in our study and found a maximum load to failure of 245 N.

The anchors in all of the aforementioned studies, according to the authors, were inserted following the manufacturers' recommendations, that is, it was inferred that they were aligned as in our group 2. The maximum loads to failure were comparatively much lower than those found in this study. This finding may be due to the fact that the tests were carried out in human cadavers, whereas in this study, ovine humeri were used. Another possible reason for this difference in maximum load to failure is that the load used for the cyclic tests was not the same, besides the difference in the number of cycles; thus, a direct comparison between the data could not be made because the test conditions were not the same.

The motivation for the biomechanical tests carried out relied on the need to investigate whether the anchor design and the shape of the eyelet would have any impact on the anchor’s biomechanical failure. Our results showed that, when selecting an anchor, the shape of the eyelet, as well as its final position in the greater tuberosity, does not need to be considered.

### Limitations

There are some limitations in this study that should be pointed out. First, only 1 anchor model was tested, so only 1 eyelet design was evaluated by the tests. Another limitation is that only the bone-anchor-suture interface was evaluated, without evaluation of the interface between the suture and the rotator cuff tendon. Because this is a biomechanical study, we cannot extend the results to the in vivo situation and determine the clinical usefulness. Additionally, the tests were conducted in nonhuman specimens, which could alter the final biomechanical and biological parameters. This is a practical way to perform biomechanical testing but is not ideal. The tests were conducted in an immediate postoperative scenario that does not reproduce the real scenario. In a real scenario, it is expected that the healing will take place prior to repetitive cyclic loading and the eyelet will be softened with scars and other biological tissues. Therefore, it is expected that the severe friction between the anchor metal eyelet and the FiberWire will be gone or lessened. Finally, the anchors were tested in only 2 alignments, perpendicular to each other. For future research, it may be necessary to extend the tests to a greater number of anchors and with different alignments.

## Conclusions

For metallic screw-in suture anchors, the alignment of the eyelet does not change the failure mode and the load to failure after cyclic loading of the bone-anchor-suture system in ovine humeri.

## Supplementary Data

ICMJE author disclosure forms
